# Physical and Genetic Structure of the Maize Genome Reflects Its Complex Evolutionary History

**DOI:** 10.1371/journal.pgen.0030123

**Published:** 2007-07-20

**Authors:** Fusheng Wei, Ed Coe, William Nelson, Arvind K Bharti, Fred Engler, Ed Butler, HyeRan Kim, Jose Luis Goicoechea, Mingsheng Chen, Seunghee Lee, Galina Fuks, Hector Sanchez-Villeda, Steven Schroeder, Zhiwei Fang, Michael McMullen, Georgia Davis, John E Bowers, Andrew H Paterson, Mary Schaeffer, Jack Gardiner, Karen Cone, Joachim Messing, Carol Soderlund, Rod A Wing

**Affiliations:** 1 Arizona Genomics Institute, University of Arizona, Tucson, Arizona, United States of America; 2 Department of Plant Sciences, University of Arizona, Tucson, Arizona, United States of America; 3 BIO5 Institute, University of Arizona, Tucson, Arizona, United States of America; 4 Division of Plant Sciences, University of Missouri, Columbia, Missouri, United States of America; 5 Plant Genetics Research Unit, Agricultural Research Service, United States Department of Agriculture, Columbia, Missouri, United States of America; 6 Arizona Genomics Computational Laboratory, University of Arizona, Tucson, Arizona, United States of America; 7 Plant Genome Initiative at Rutgers, Waksman Institute, Rutgers, The State University of New Jersey, Piscataway, New Jersey, United States of America; 8 Plant Genome Mapping Laboratory, Departments of Crop and Soil Science, Plant Biology, and Genetics, University of Georgia, Athens, Georgia, United States of America; 9 Division of Biological Sciences, University of Missouri, Columbia, Missouri, Arizona, United States of America; The Salk Institute for Biological Studies, United States of America

## Abstract

Maize *(Zea mays L.)* is one of the most important cereal crops and a model for the study of genetics, evolution, and domestication. To better understand maize genome organization and to build a framework for genome sequencing, we constructed a sequence-ready fingerprinted contig-based physical map that covers 93.5% of the genome, of which 86.1% is aligned to the genetic map. The fingerprinted contig map contains 25,908 genic markers that enabled us to align nearly 73% of the anchored maize genome to the rice genome. The distribution pattern of expressed sequence tags correlates to that of recombination. In collinear regions, 1 kb in rice corresponds to an average of 3.2 kb in maize, yet maize has a 6-fold genome size expansion. This can be explained by the fact that most rice regions correspond to two regions in maize as a result of its recent polyploid origin. Inversions account for the majority of chromosome structural variations during subsequent maize diploidization. We also find clear evidence of ancient genome duplication predating the divergence of the progenitors of maize and rice. Reconstructing the paleoethnobotany of the maize genome indicates that the progenitors of modern maize contained ten chromosomes.

## Author Summary

As a cash crop and a model biological system, maize is of great public interest. To facilitate maize molecular breeding and its basic biology research, we built a high-resolution physical map with two different fingerprinting methods on the same set of bacterial artificial chromosome clones. The physical map was integrated to a high-density genetic map and further serves as a framework for the maize genome-sequencing project. Comparative genomics showed that the euchromatic regions between rice and maize are very conserved. Physically we delimited these conserved regions and thus detected many genome rearrangements. We defined extensively the duplication blocks within the maize genome. These blocks allowed us to reconstruct the chromosomes of the maize progenitor. We detected that maize genome has experienced two rounds of genome duplications, an ancient one before maize–rice divergence and a recent one after tetraploidization.

## Introduction

Cereal crops, such as rice, maize, and wheat, are the major caloric source for humans and farm animals. The cereals shared a common ancestor some 50 million years ago (MYA) and even though their genome sizes vary considerably, their genetic map organization is highly conserved [1,2]. Rice (genome size = 389 Mb [3]) was the first cereal to have its genome completely sequenced and now serves as a reference sequence for comparative and functional genomics studies across the cereals. The 2,300-Mb [4] maize B73 genome is presently being sequenced by a clone-by-clone sequencing strategy. Although the maize genome behaves genetically as a simple diploid with ten pairs of chromosomes, its organization is quite complex. Early genetic analysis of duplicated genes suggested that maize had homoeologous regions [5–7]. This work was later supported by comparative restricted fragment length polymorphism (RFLP) mapping across the cereals, which showed that two maize chromosome sets aligned with one chromosome each of rice and sorghum, thereby demonstrating a whole genome duplication event [1,8]. Evolutionary analysis of duplicated genes indicated that maize may have arisen by allotetraploidy, suggesting that maize was formed by the hybridization of two slightly diverged progenitors rather than the duplication of a single progenitor [9]. While cytogenetic studies suggested that the progenitors of maize were species consisting of five chromosomes [10], alignments of linkage maps of rice and maize hypothesized that the progenitors had eight chromosomes [11]. Furthermore, these maps also indicated that the ancient duplications in rice were also present in the progenitors of maize, suggesting that the duplications occurred before the split of the progenitors of rice and maize [12–15], and it is likely that they would be shared by most if not all cereals [16].

Based on recent alignments of orthologous regions of rice and sorghum chromosomes with two homoeologous regions in maize, the progenitor of sorghum and the two progenitors of maize diverged at nearly the same time about 11.9 MYA. Furthermore, the two progenitors of maize may have hybridized as recently as 4.8 MYA, explaining the divergence of the duplicated regions in maize [17]. Alignments of chromosomal regions shared by common ancestry also showed that frequently one copy of the duplicated genes was lost [18,19].

Sequence analysis of random sheared fragments [20], BAC (bacterial artificial chromosome) end sequences (BESs) [21], and complete BAC sequences from 100 random regions [22] showed that about two-thirds of the maize genome consists of transposable elements, with 95% of those being retrotransposons. Because long terminal repeats are identical at the time of insertion, it was possible to determine the phase of expansion. It appears that retrotranspositions emerged after speciation of maize with the majority of elements occurring less than 1 MYA [23].

Genome size, reduplication, and high repetitive DNA content make maize an extremely challenging genome to sequence, even with the availability of the rice genome as a reference sequence. Sequencing projects using a clone-by-clone strategy, as well as many whole genome shotgun projects, require the availability of a high-resolution physical map. Here, we present the final assembly and analysis of the physical map of Zea mays ssp. *mays* cv. B73, which is comprised of many complementary resources including: (1) deep-coverage large insert BAC libraries [24,25]; (2) agarose and high information content fingerprint (HICF) datasets assembled with fingerprinted contig software (FPC) [26,27]; (3) a genetic marker dataset to anchor the physical map to the maize genetic map; and (4) sequence-based marker datasets (overgos [28] and BESs [21]) to infer contig position and orientation with respect to the rice reference sequence [3]. Alignment of the two genomes allowed us to shed new light on the organization of the chromosomes of the progenitors contributing to the maize polyploidization, unravel rearrangements associated with the subsequent diploidization of the maize genome, and infer the sizes and locations of homoeologous regions together with spatial patterns of genome size evolution. This also sets the stage for careful comparison of maize to sorghum and to begin to dissect the shared versus lineage-specific consequences for these genomes of an ancient duplication common to all cereals.

## Results/Discussion

### Integrated Physical and Genetic Map of the Maize B73 Genome

A total of three deep-coverage large-insert BAC libraries covering ∼30 genome equivalents were constructed using HindIII, EcoRI, and MboI digests of high molecular weight DNA isolated from maize inbred line B73 [24,25]. We used two different methods to fingerprint the same set of BAC clones, referred to as agarose [29] and HICF [30,31], to cross-confirm the assembled physical contigs of homoeologous regions and highly conserved sequence families. The agarose method resulted in 292,201 successful fingerprints that were automatically assembled into 4,518 contigs using FPC [26] at a Sulston score of 1e−12 and tolerance of seven (Table S1). HICF generated 350,253 successful fingerprints that automatically assembled into 1,500 FPC contigs [27]. This was accomplished by assembling the initial contigs using a Sulston score of 1e−70 and then automatically merging contigs with a Sulston score of 1e−21.

A total of 25,908 markers were integrated into the FPC map that included 1,902 genetic markers and 24,006 overgo/BES markers. In addition to providing valuable genetic and biological information, these markers were also extremely useful in the validation of FPC contig assemblies. A total of 2,036 genetic markers including 1,307 SSRs (simple sequence repeat), 372 RFLPs, 189 SNPs, and 168 insertion/deletions were used to integrate the FPC map with the maize genetic map (Table S2). After integration, 1,902 genetic markers (93.4%; 1,240 SSRs, 345 RFLPs, 169 SNPs, and 148 insertion/deletions) could be placed confidently on the physical map (Table 1; Table S3 for the distribution of these markers). These included 822 well-ordered IBM (intermated B73 × Mo17) framework markers (94.6% success rate) and 1,080 binned IBM-neighbor placement markers (see Methods for marker definitions; 92.5% success rate) (Table S2).

**Table 1:**
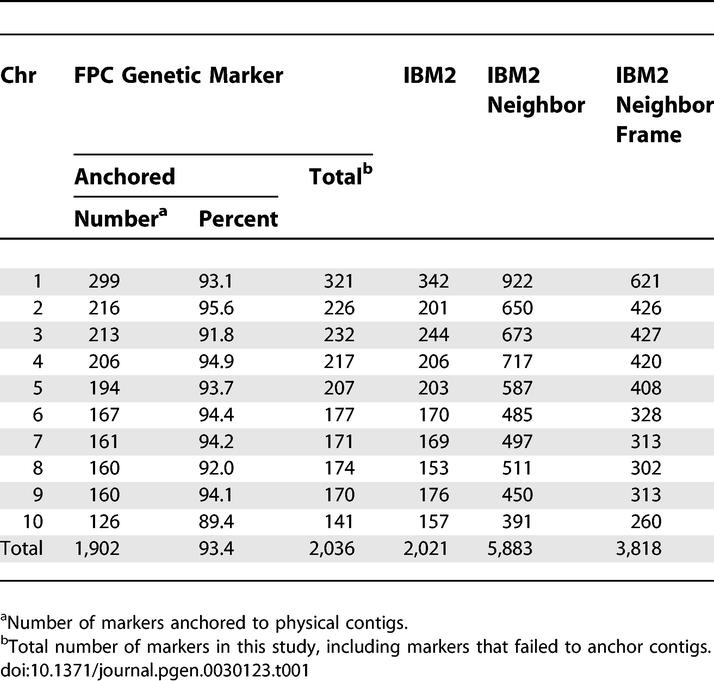
Maize Genetic Markers

In addition to the genetic markers, 24,006 additional sequence-based markers were integrated into the maize FPC map. Although these markers were not genetically mapped, they proved to be extremely powerful by providing sequence tag sites that could be used to order and orient contigs relative to the rice reference sequence [3]. Sequence-based markers included 3,438 privately contributed EST (expressed sequence tag)-derived unigene markers, 9,371 overgos derived from 70,716 maize ESTs [28], 2,068 highly conserved cereal sequences, and 9,129 gene-containing BESs [21]. As shown in Table 2, all marker types appear to be distributed across the maize chromosomes roughly in proportion to the sizes of the respective chromosomes.

**Table 2:**
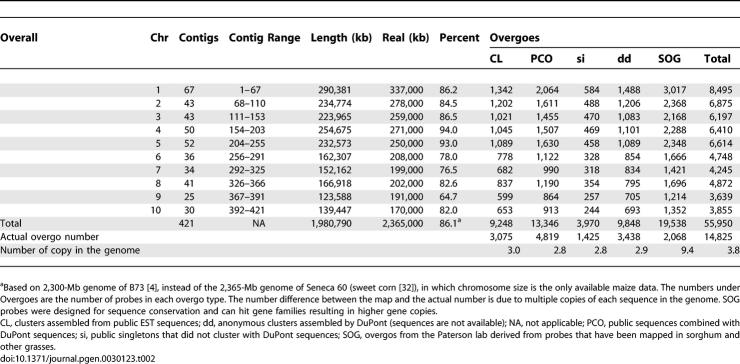
Summary of the Maize Physical Map

In manual editing of the agarose physical map, four sources of evidence were assessed to indicate contig placement, orientation, or merging—the agarose FPC map, markers, the HICF FPC map, and synteny between maize and rice (see below). Only if evidence from three of the four criteria was met would two contigs be merged. The final FPC map resulted in 721 contigs covering 2,150 Mb (equal to 93.5% of the 2,300-Mb genome; see Methods for detailed calculations).

Of the final 721 contigs, 421 are anchored to the maize genome using 1,902 genetic markers. The anchored contigs cover 1,981 Mb, equal to 86.1% of the maize genome (Table 2). Of the remaining 300 unanchored contigs (7.4% of the genome), 189 contain fewer than ten BAC clones each. The average sizes of anchored and unanchored contigs were 4.7 Mb and 0.56 Mb, respectively. The longest anchored contig was 22.9 Mb on Chromosome 9 (chr9), while the longest unanchored contig was 6.7 Mb. The individual chromosome coverage varied from 94% on chr5 to 65% on chr9. The low coverage found on chr9 does not, perhaps, reflect the real situation due to the fact that 94.1% of the genetic markers could be placed on the FPC map for this chromosome. This discrepancy could possibly be explained by genome size disparity found in various maize cultivars, such as differences in heterochromatic regions. The chromosome sizes used here, the only available maize data, were derived from sweet corn (Seneca 60) [32]. The fact that the largest anchored contig is on chr9 (22.9 Mb) may indicate that maize chr9 in B73 is highly euchromatic.

The average physical to genetic ratio in the maize genome is 182 kb per cM with great variation, ranging from >1.8 Mb/cM in centromeric regions to <10 kb in telomeric region. The rich marker information integrated into the physical map allowed us to plot marker distribution along the maize chromosomes. When compared with genetic marker distribution (Figure S1), we found that EST-derived markers are directly correlated with genetic markers, indicating a tight association of gene distribution and genetic recombination.

The maize physical map can be accessed at http://www.genome.arizona.edu/fpc/maize.

### Maize–Rice Syntenic Block and Its Implications

**Figure 1:**
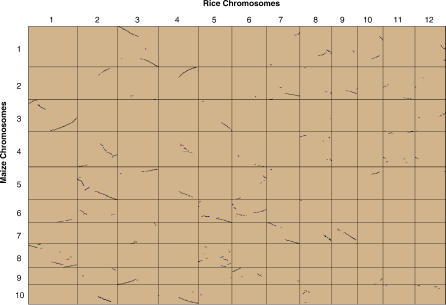
Dotplot Analysis of the Integrated Maize Map against Rice Pseudomolecules Synteny blocks were detected, and background noise was filtered with SyMAP [37]. The interactive dotplot can be viewed at http://www.agcol.arizona.edu/symap. When clicking the related synteny block, the detailed window with contig number will pop up. The viewer can select the preferred area and double click the selection, and then a graphic alignment is displayed.

Similarities in gene order between rice and maize have been extensively reported [1,11,15,33–36]. However, these studies used low-resolution genetic maps or incomplete rice sequences. Now, using the rich marker information embedded in our integrated maize physical map and the rice reference sequence [3], we were able to build a high-resolution comparative physical map between the maize and rice genomes. Using synteny mapping and analysis program (SyMAP) software [37] we generated a dotplot between the integrated maize physical map and the 12 rice pseudomolecules and then computed syntenic blocks (see Methods) with consideration of marker density and position within the integrated maize map (Figure 1). As shown in Table S4, 52 major maize–rice collinear blocks could be identified. Previous studies suggested 20 chromosomal rearrangements in maize compared to rice but could not provide accurate sizes of syntenic blocks because of the paucity of markers [11]. These 52 maize blocks varied in size from 760 kb to over 82 Mb, and their corresponding rice regions fluctuated from 360 kb to over 22 Mb.

**Figure 2:**
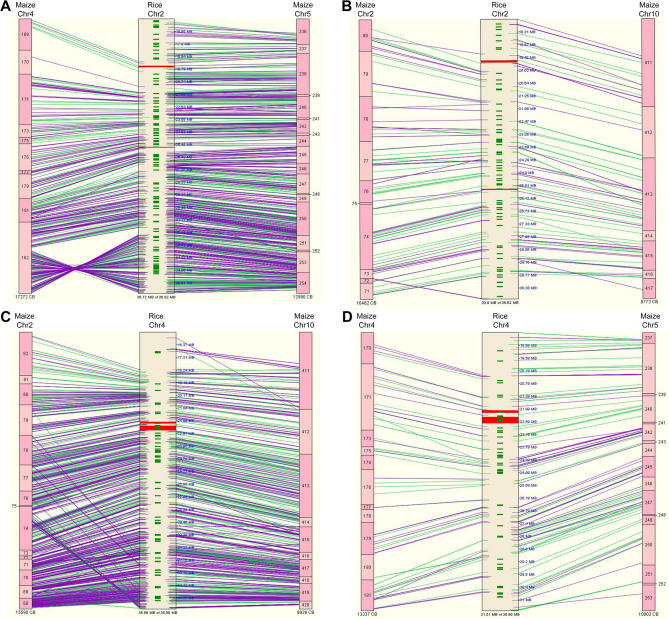
Graphic Display of Two Rounds of Maize Genome Duplication The picture was captured from the comparative block display of the integrated maize–rice synteny map generated by SyMAP [37]. Green alignment line showed result from overgo markers, and violet line showed alignment from low-copy BES. (A) Recent duplication resulted from ditetraploidization of maize chr4L and 5L with reference to rice 2L. (B) Ancient duplication before maize and rice divergence of maize chr2S and chr10L with reference to rice 2L is presented. (C and D) show that with reference to rice chr4L, recent and ancient duplications have occurred of maize chr2S and chr10L, and chr4L and chr5L, respectively.

Although the maize genome is about 6-fold larger than rice, in collinear regions on average, every kilobase of rice sequence expands to only about 3.2 kb of sequence in maize, with a range of 1.1–9.5 kb in different regions (Table S4). This uneven expansion of syntenic blocks appears to be due to differential loss of genic regions and insertion of retrotransposable elements as recently shown by sequence comparison of a pair of homoeologous regions of maize chr1S and chr9L with rice chr3S [18]. Since on average one region in rice corresponds to two maize regions, one would expect the expansion factor for each maize region to be half of the total genome expansion. Therefore, the derived factor of 3.2 is consistent with the whole genome duplication event described in Figure 2. The maize sytenic regions (1,474.5 Mb in total) cover 74.5% of the anchored map (64% of the genome). The regions in Figure S2A that do not show maize–rice correspondence appear to be heterochromatic, also as reported for the sorghum–rice comparison [38].

An interactive display of the rice-maize synteny is available at http://www.agcol.arizona.edu/symap.

### Genome Duplication and Genome Rearrangement during Maize Evolution

From the dotplot in Figure 1, we observed that in addition to the primary syntenic blocks in rice (Table S3) each maize syntenic block corresponds to another syntenic region in rice, consistent with previous reports [12–15] that used translated protein sequences to show ancient duplications in rice, which were phylogenetically dated before the divergence of most if not all cereals [16]. Whereas they used rice-to-rice comparisons, we used the maize alignment to rice to detect synteny blocks that elucidated the rice duplication.

The advantage of comparing rice to maize instead of rice to rice is that all ancient duplications that predate the ancestor of rice and maize should also be present in the progenitors of maize. Therefore, if maize arose from two progenitors by allotetraploidization, segmental duplications should be present in four copies. Indeed, our high-resolution integrated physical map shows examples of such segmental quadruplications and allows us to more precisely determine their coordinates. Using the rice genome as a reference to investigate the maize genome, we found that each syntenic region in rice has three to four corresponding regions in maize (Figures 1 and S2; Table 3). The orthologous maize–rice regions (primary blocks) have higher density synteny alignments than the “paleologous” regions resulting from ancient duplications (secondary blocks). Figure 2A shows the primary synteny of the long arm of rice chr2 with maize chr4 and chr5, and Figure 2B shows secondary synteny of the same rice chr2 region with maize chr2 and chr10 (see also Figure S2B). Figure 2C and 2D shows that the long arm of rice chr4 is primarily syntenic with maize chr2 and chr10 and secondarily syntenic to maize chr4 and chr5 (also see Table 3). Figures 1 and S2A show that maize chr2, chr4, chr5, and chr10 all align to the same region of rice chr2 and chr4. These results are consistent with previous reports that showed the long arms of rice chr2 and chr4 to be syntenic to one another [12,13,15]. Table 3 summarizes all the primary and secondary duplication blocks in maize on the basis of our analysis. In addition, the detection of an average of around three copies for each overgo (Table 2) further supports the idea that the present maize genome emerged from tetraploidization of several ancient duplicated regions. On the other hand, formation of duplications in the rice genome is not limited to the ancestor of rice and maize. The most recent segmental duplication in rice of 3 Mb on the tips of rice chr11 and chr12 is estimated to be about 7.7 MYA [39], well after rice–maize divergence. This illustrates a general model that small segmental duplications (such as the 7.7 MYA one) continue to arise over long periods of time unlike a whole genome duplication event and therefore contribute to the uniqueness of each genome [14,39]. Multiple duplication events may also contribute to the maize-complex duplication pattern reported previously [11,40], in which the recent maize duplication is much easier to be identified than the ancient genome duplication in a cereal ancestor.

**Table 3:**
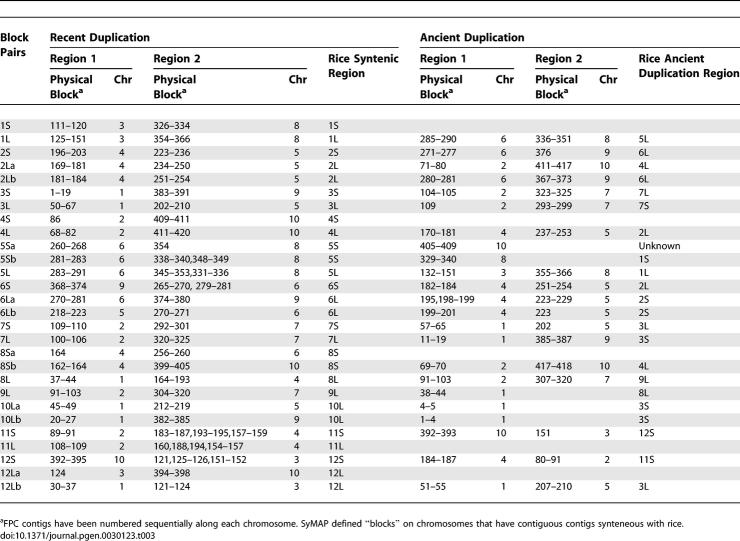
Two Genome Duplication Events in Maize Genome Evolution

Our synteny analysis also shows that several genome rearrangements have occurred since the divergence of maize and rice. As shown in Table S5, we identified 62 rearrangements (>125 kb in corresponding rice sequence) across all chromosomes, which account for 281.2 Mb (19%) of the maize 1,474.5 Mb syntenic sequence with a block size variation from 440 kb to over 32 Mb. These rearrangements include 39 inversions (207.2 Mb), 14 inversion and translocation events (56.7 Mb), and eight translocations (16.3 Mb). Most of these rearrangements were detected near telomeres. A total of 12 of the 20 maize telomeric ends or 15 of 24 rice telomeric ends have undergone rearrangements in one way or the other; most inversions may be due to high recombination frequency in the regions. It is rare to find interchromosomal rearrangement, supporting the role of recombination in these macrorearrangements. Although the majority of rearrangements can be confirmed using our integrated map, it is possible that a few may have resulted from fingerprint or sequence misassembly in maize or rice, respectively. The maize genome-sequencing project as well as rice and maize optical maps will likely resolve such discrepancies in the near future.

### Chromosome Reconstruction of Maize and Its Ancestors

The 52 major maize–rice collinear blocks (Table S4) and 28 maize duplication block pairs (Table 3) allow us for the first time to demonstrate the extent of chromosome breakage and fusion that occurred in the maize genome after chromosome doubling from its two progenitors. Furthermore, with the assistance of the rice reference sequence [3], we can propose a model for the chromosome structure of the progenitors of maize and their ancestors.

**Figure 3:**
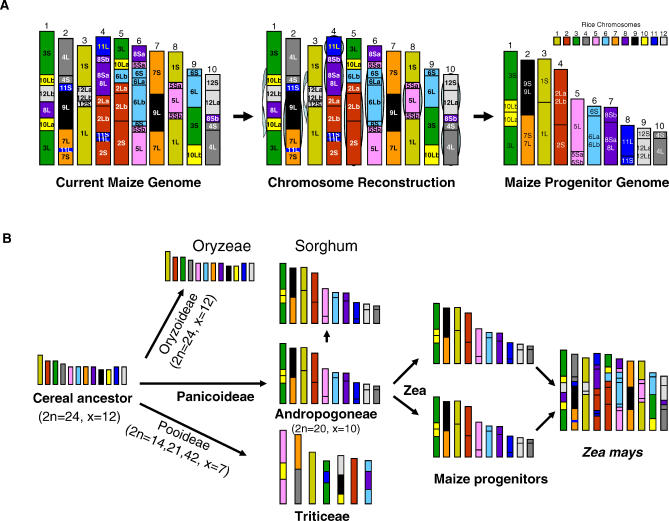
Change of Chromosome Numbers during Speciation of Cereals Based on the dotplot comparison of rice, maize, sorghum, and wheat chromosomes, synteny blocks have been used to assemble progenitor chromosomes of these species. Rice synteny blocks have been color coded. (A) Using the rice color-code sytenic block from Table 3 and Table S4, the chromosomes of the progenitors of maize have been reconstructed. The block names in the figure are the same in Table 3. No change in chromosome number occurred, but an increase of maize chromosome sizes did. (B) Comparison of the relationship of the maize progenitors with sorghum and wheat has been used to reconstruct the changes and conservation of chromosomes during speciation.

When the maize homoeologous regions are reassembled into homoeologous chromosome pairs and are then aligned with homologous rice chromosomes (Figure 3A), a picture emerges that the two progenitors of maize each consisted of ten chromosomes resulting in ten chromosomes after hybridization. The most parsimonious explanation is that the progenitor chromosomes underwent breakage and fusion and eventually reassembled into ten new mosaic chromosomes that are genetically diploid as opposed to being allotetraploid. Therefore, it is most likely that the syntenic blocks discovered here reflect the new junctions that were formed in this process. One possible explanation for the formation of a 2*N* = 20 diploid, composed of reshuffled chromosomes rather than a 2*N* = 40 allotetraploid after hybridization, is that maize lacks the equivalent of the *Ph*1 product/structure that prevents pairing of homoeologous chromosomes in wheat [41]. There are examples where allopolyploidy does not result in reshuffling of chromosomes as in the case of polyploid wheat. It is interesting to note that the *Ph*1 locus in wheat prevents the pairing of nonhomologous chromosomes. One can envision that the lack of such a function in maize has resulted in alignments of nonhomologous chromosomes after allotetraploidy and contributed at least in part to their rearrangements. In addition, a burst of transpositions of transposable elements or ectopic recombination or conversion might have triggered chromosome breakages and fusions on a larger scale. However, these are just possible scenarios, and perhaps sequences of multiple inbred lines could be more informative about the possible mechanisms.

When compared to the rice–sorghum synteny, the ten maize progenitor chromosomes appear to be the same as the ten sorghum linkage groups (Figure 3B). After divergence from rice, the common ancestor of maize and sorghum combined rice chr3 and chr10 to form one chromosome and rice chr7 and chr9 to form another chromosome. This resulted in ten chromosomes in the maize and sorghum ancestors (Figure 3A). Using a more limited dataset, Wilson et al. [11] proposed that in addition to the above chromosome fusions, two additional fusions occurred—one between rice chr1 and chr5 and the other between rice chr4 and chr12—hypothesizing an eight-chromosome model for the progenitors of maize. Our data did not support these two fusions. By comparison of this rice–Andropogoneae synteny to synteny between rice and wheat [42], we found there are no common chromosome combinations with respect to the rice genome after divergence from the common ancestor. These data suggest that the rice genome may represent the ancient ancestral form of cereal genomes (Figure 3B). Clearly, the high-density gene map of maize has raised the level of our understanding of the role of speciation in the chromosome evolution in plants.

## Methods

### DNA fingerprinting and band analysis.

The three BAC libraries (30× total coverage) used in this study were HindIII (136-kb average insert size), EcoRI (163-kb), and MboI (167-kb) libraries [24,25], with 14.2×, 7.6×, and 7.8× coverage respectively. The use of three BAC libraries helped to ensure the maize genome was fully represented. Maize cultivar B73 was selected because of its widespread use in breeding and because it is one of the parents of a public high-resolution genetic reference mapping population (IBM [43]).

BAC clones starting with a “b” are from the B73 HindIII library (ZMMBBb). BAC clones starting with a “c” are from the B73 EcoRI and MboI libraries known as CHORI-201 (Clemson University Genomics Institute/Arizona Genomics Institute [CUGI/AGI] name ZMMBBc). In the c library, the first half (288 384-well plates) was from the EcoR1 digest, and the second half was from the MboI digest. Clones ending in “sd1” are sequenced BACs downloaded from GenBank (http://www.ncbi.nlm.nih.gov/Genbank) digested with HindIII in silico [44]. If the sequences were longer than 150 kb, they were artificially fragmented into multiple overlapping clones, i.e., sd1, sd2, etc. and then digested with HindIII in silico. For the agarose method [29,45], all BAC DNAs were digested with the restriction enzyme HindIII, run on high-resolution 1.0% agarose gels, stained with SYBR Green (FMC BioProducts, http://www.fmc.com), and the Image software [46] was used for interactive band calling.

For the HICF method [27,30,31], BAC DNAs were digested with type IIS restriction enzyme EarI, in which the resulting ends were tagged with fluorescently labeled ddNTPs and TaqI, which is to reduce the fragment sizes of the EarI digestion. The first base in a two-base overhang from TaqI is G, therefore ddCTP was not used in the end-labeling reaction because the majority of fragments would contain at least one TaqI site. Thus we only used C, T, and A overhangs for end labeling by using the fluorescent dyes ddGTP (blue), ddATP (green), and ddTTP (yellow), respectively. Red dye (GeneScan-500 ROX, Applied Biosystems, http://www.appliedbiosystems.com) was employed as an internal size standard, which was run in each capillary. Reaction products were resolved on ABI3700 automated DNA sequencers. ABI GeneScan version 3.7.1 software (Applied Biosystems) was used for band detection and extraction. Only fragments ranging in size from 75 to 500 bp were used for HICF assembly. Because FPC can only input one set of band values for each clone, we used the technique of Ding et al. [31] to convert the size/color pairs generated by HICF. Each band was multiplied by 20, and the fractional part discarded. An offset was then added to each band as follows: 0 to blue, 10,000 to green, and 20,000 to yellow bands. Since we used fragments in the range 75–500 bp, the result of this conversion was bands occupying the ranges 1,500–10,000, 11,500–20,000, and 21,500–30,000.

### Contig assembly and manual editing.

FPC software [26] was used for fingerprint assembly. To automatically build the agarose map, two clones assemble together if they share at least *N* markers and have a Sulston score of less than *M,* using the following (*N, M*) pairs: (0, 1e−12), (1, 1e−11), (2, 1e−10), or (3, 1e−09). False-positive overlaps generally result in a stack of clones within the contig whose bands do not align well with the underlying consensus band map; these clones are called Q (questionable) clones [47]. The FPC DQer function reassembles all contigs that have >5 Q clones using a more stringent cutoff, such as 1e−13, 1e−14, or 1e−15. If the more stringent cutoff breaks the contig into multiple contigs, the function tries to join the subcontigs by their end clones; if this is not possible, it creates new contigs from the subcontigs. If it cannot reduce the number of Qs by a 1e−15, it leaves the contig alone. Generation of the maize FPC map was performed in stages including manual editing that utilized the agarose assembly of 4,518 contigs and all genetic and overgo markers. The addition of these markers allowed us to reduce the Sulston score for contig merging. Singletons were added in to merge contigs when they overlapped with the ends of two contigs. The total contig number decreased from initial build of 4,518 to 2,085, and the assembly was publicly released in February 2004 (Table S1).

To automatically build the HICF map, possible contaminated clones were screened for same-plate overlaps at 1e−45, followed by removal of all remaining clones over 175 bands. The initial contigs were built at 1e−70 at the tolerance of 4, then the DQer reassembled all contigs that had >15% Q clones. The end-merging function was run at 1e−61, 1e−52, 1e−45, 1e−40, and 1e−21, where the end-merging function checks the clones at the ends of contigs, and if at least two pairs of clones overlap based on the given cutoff, the contigs are merged. Singletons were placed at 1e−43 to their best location, before the final end-merge.

The agarose map was edited to split contigs with false-positive joins and to merge contigs that had overlapping end clones that were too small to be detected by FPC. First, manual end joining was performed if the end clones from two contigs had a Sulston score of *N* and at least *M* shared markers, where the valid (*N, M*) pairs were (0, 1e−10), (1, 1e−09), (2, 1e−08), or (3, 1e−07). We also extensively used the HICF map, marker information, and maize–rice synteny for contig merging. A new marker type called HICF was created, which was used to link each pair of clones that are bridged by an HICF clone that did not occur in the agarose map. We know that the pair of clones lie close to each other (within 150 kb) and probably overlap, but it cannot be confirmed. The agarose method produced an average of 30 bands for each maize clone (band size range from 1 to 23 kb), while the HICF method generated an average of 107 bands for each clone (size range 75 bp–500 bp [27]).

The metric of a FPC contig is the consensus band (CB) unit. If the length of a contig is *N* CB units, then its approximate length in base pairs is *N* × 4,900. The 4,900 was calculated as the average size restriction fragment as follows: A total of 20 completely sequenced maize BACs that had corresponding fingerprints were downloaded from GenBank. The average size of all sequenced BACs was divided by the average number of fingerprinted bands, which resulted in an average band size of 4,900 bp. Since the number of bands was taken from the real fingerprint and not from the simulated fingerprint, this average size took into account missing bands. This estimation is similar to that in the rice physical map [45].

### Genetic mapping.

As an essential component to establish an integrated map for the maize genome, a saturated and evenly distributed genetic map was developed to anchor and fully orient physical contigs along the chromosome. We built a dense genetic framework using the IBM mapping population [43]. The inbred parents of IBM are highly polymorphic and the progeny experienced four rounds of intermating, which makes IBM nearly four times expanded in size and 18-fold higher in resolution than previous public standard populations [48]. The order of loci on the IBM map has proven to match closely that developed by fingerprinting and BAC assembly.

The number of genetically mapped markers was increased by intercalating locations of additional markers into the IBM, constructing “neighbors'” maps (Table 1), i.e., by importing markers from maps of other mapping populations and interpolating for genetic coordinates [49]. Successive iterations of neighbors' maps have incorporated new map data from other projects and have refined orders according to FPC results. Maps have been updated regularly in MaizeGDB [50].

### Integration of genetic markers with the FPC map.

We used probe hybridization on BAC filters, primer amplification methods on 6-D BAC pools, and overgo probe hybridizations to integrate genetically mapped markers with the related BAC clones in the maize physical map. Since genetic markers can hybridize to multiple contigs because of paralogous sequences, it was necessary to distinguish between marked contigs that could be genetically placed and all other copies. To this end, anchoring markers retained their original name, and any unanchored copies were assigned a suffix (.A). Markers included RFLPs, SSRs, ESTs, SNPs, and InDels. Filter hybridization has low throughput, so we used only 90 RFLP probes on filters [24] and carried out further integrating with two high-throughput methods.

Hybridization of overgos was conducted on four filters of a 6 × 6 grid with a total of 165,888 BACs, half from each of the HindIII and EcoRI libraries. The 40-bp overgo probes were designed from unigenes of the DuPont/MMP/Incyte Genomics partnership and were screened with a maize repeat database to ensure that each probe was low copy. The overgos were classified into five marker types where the prefix identifies the marker type: (1) the prefix CL identifies clusters assembled from public EST sequences; (2) PCO represents public sequences combined with DuPont sequences (Unigene Consensus deposited in GenBank); (3) dd signifies anonymous clusters assembled by DuPont (sequences are not available); (4) si represents public singletons that did not cluster with DuPont sequences; and (5) SOG markers are overgos from the Paterson lab, derived from probes that have been mapped in sorghum and other grasses. Except for the SOG markers, which were designed for sequence conservation, all other overgos were designed for their diversity. We employed a 2-D 24 × 24 pool strategy for overgo hybridization and scored dual grid spots as positive clones [28].

PCR methods were used to screen BACs with SSR or genomic sequence markers. A 6-D pool strategy [51] was used for library screening to construct 288 pools from a 48 × 48 × 48 block for a total of 82,944 BACs in 216 384-well plates from the HindIII library. Primers for selected sequences were applied to pools, and the data were deconvoluted using criteria that assured disambiguation by multiple confirmation.

### Maize–rice synteny analysis.

We used the SyMAP [37] to compute and view the syntenic blocks. The algorithm first computed anchors from the alignment of the maize markers and BES sequences to the rice genomic sequence and then calculated chains of anchors using dynamic programming and merged chains into blocks. The graphical results from a versatile CGI/HTML and Java Display allow users to observe synteny from different views, including dotplot, genome blocks, chromosome blocks, close-up chromosomes, close-up contigs, and table view. The results can be interactively browsed at http://www.agcol.arizona.edu/symap.

## Supporting Information

Figure S1 Correlation between Gene Distribution and Genetic Recombination The distribution of EST-derived overgo markers and genetic markers was plotted against maize chr1. The overgos, which hybridized to at least two BAC clones in each contig and hit fewer than ten contigs, were counted for the plot. The overgos and genetic markers were counted as a total in each Mb region.
(44 KB PPT)

Figure S2 Overall Block View of Maize–Rice Synteny (A) General view of maize–rice synteny alignment is presented. This is the block view generated from dotplot analysis in Figure 1. Rice chromosomes are in the middle of each synteny block as gray vertical bars, and rice centromeres are shown in red. Maize chromosomes are colored as shown in the horizontal color key.
(B) Physical synteny map of rice chr2 with maize chromosomal regions is presented. Each maize physical block is indicated by chromosome number and FPC contig numbers. When clicked in our interactive website (http://www.agcol.arizona.edu/symap), an individual block view with rice alignment or a comparative block view like Figure 2 will pop up.
(72 KB PPT)

Table S1 Major Release of the Maize Physical Map (16 KB XLS)

Table S2 Summary of the Maize Genetic Map (26 KB XLS)

Table S3 Distribution of Different Marker Types among Chromosomes (16 KB XLS)

Table S4 Primary Synteny of Maize and Rice (30 KB XLS)

Table S5 Chromosomal Fragment Rearrangement after Maize–Rice Divergence (31 KB XLS)

## Abbreviations

BAC: bacterial artificial chromosome
BES: BAC end sequence
chr: chromosome
EST: expressed sequence tag
FPC: Fingerprinted contig
HICF: high information content fingerprint
IBM: intermated B73 × Mo17
MYA: million years ago
RFLP: restricted fragment length polymorphism
SSR: simple sequence repeat
SyMAP: synteny mapping and analysis program
